# Autophagy gene expression profiling identifies a defective microtubule-associated protein light chain 3A mutant in cancer

**DOI:** 10.18632/oncotarget.9754

**Published:** 2016-05-31

**Authors:** Joana R. Costa, Krisna Prak, Sarah Aldous, Christina Anja Gewinner, Robin Ketteler

**Affiliations:** ^1^ MRC Laboratory for Molecular Cell Biology, University College London, London, United Kingdom; ^2^ Translational Research Office, University College London, London, United Kingdom

**Keywords:** ATG4B, autophagy, gene expression, luciferase release assay, MAP1LC3A R70H

## Abstract

The cellular stress response autophagy has been implicated in various diseases including neuro-degeneration and cancer. The role of autophagy in cancer is not clearly understood and both tumour promoting and tumour suppressive effects of autophagy have been reported, which complicates the design of therapeutic strategies based on targeting the autophagy pathway. Here, we have systematically analyzed gene expression data for 47 autophagy genes for deletions, amplifications and mutations in various cancers. We found that several cancer types have frequent autophagy gene amplifications, whereas deletions are more frequent in prostate adenocarcinomas. Other cancer types such as glioblastoma and thyroid carcinoma show very few alterations in any of the 47 autophagy genes. Overall, individual autophagy core genes are altered at low frequency in cancer, suggesting that cancer cells require functional autophagy. Some autophagy genes show frequent single base mutations, such as members of the ULK family of protein kinases. Furthermore, we found hotspot mutations in the arginine-rich stretch in MAP1LC3A resulting in reduced cleavage of MAP1LC3A by ATG4B both *in vitro* and *in vivo*, suggesting a functional implication of this gene mutation in cancer development.

## INTRODUCTION

Autophagy is a highly conserved process that serves two main functions in the cell: to provide energy under conditions of limited nutrient availability and to remove damaged organelles such as misfolded protein aggregates or non-functional mitochondria from the cell. As a consequence, malfunction of this process has severe consequences that contribute to various diseases including pathogen infection, neuro-degenerative disorders and cancer [1]. In addition, studies suggest that autophagy is contributing to the ageing process of an organism [2], a feature that may be linked to increased cancer occurrence in aged cells. It has been suggested that autophagy plays a key role in the initiation, progression and differentiation of tumours and to enhance tumour cell survival under conditions of cellular stress and has also been postulated to serve as a tumour suppressive mechanism [3, 4].

The most compelling evidence for autophagy as a tumour suppressive mechanism is based on the finding that Beclin-1 (BECN1), a gene involved in autophagosome formation, has been found haploinsufficient in around 50% of breast, ovarian and prostate cancers [5]. Furthermore, BECN1 hemizygous mice develop lymphoma, liver and lung cancers [6, 7]. However, a more detailed analysis of sequencing data has recently suggested that loss of the neighbouring gene, BRCA1, is the primary driver alteration in cancer [8]. In addition to BECN1, frameshift mutations in core autophagy genes ATG2B, ATG5, ATG9B and ATG12 in colorectal cancers have been reported, providing additional evidence for a tumour suppressive role [9]. Furthermore, mice lacking the ZBTB24 or ATG4C gene are prone to tumourigenesis [10, 11]. Mechanistically, the tumour suppressive role has been mainly attributed to an increase of reactive oxygen species and DNA damage when autophagy is impaired [12], although other mechanisms may be relevant as well.

On the other hand, autophagy can promote tumour growth by multiple mechanisms including the suppression of p53 activity, as well as maintaining mitochondrial function and enhancing survival under conditions of stress [13]. A seminal paper in 2006 described promotion of tumour necrosis when autophagy was inhibited in apoptosis-defective cells, thus leading to the concept of autophagic cell death under conditions of metabolic stress [14]. In addition, the deletion of RB1CC, an essential autophagy gene, inhibits oncogene-driven tumourigenesis [15]. Furthermore, it has been shown that ATG4B can promote growth of CML and colorectal cancer cells [16, 17]. Since cancer cells undergo periods of nutrient limitation, it has been proposed that autophagy benefits the tumour by promoting cell survival under stress conditions.

Based on the crucial role of autophagy in tumourigenesis, treatments exploiting the autophagy pathway have been suggested. These recent clinical advances are supported by two prevailing hypotheses that are based on the important role of autophagy in stress-induced cell death. One is that cancer cells undergo phases of cellular stress, leading to an upregulation of autophagy in general. Cellular stress can occur under conditions such as hypoxia, nutrient scarcity during tumour expansion or during treatment with chemotherapeutic agents. Under these conditions, a suppression of autophagy may result in stress-induced cell death and may be therapeutically exploited. On another account, autophagy displays a tumour suppressive role as has been suggested for Beclin-1 or ATG4C. In this instance hyper-stimulating autophagy may mimic a state of cellular stress leading to a ‘point of no return’ and consequently to “autophagic cell death”. So far, evidence for an induction of autophagic cell death specifically in tumour cells is lacking and this remains a hypothesis that needs to be proven.

To date, close to 50 clinical trials have been reported to investigate the impact of autophagy inhibition using hydroxychloroquine or its derivatives, mTOR inhibitors or AKT inhibitors, usually in combination with chemotherapeutic agents (www.clinicaltrials.gov). Whether the effects of such treatments are indeed caused by modification of the autophagic pathway or alternative processes that are also targeted by such treatments is not clear, since robust autophagy assays essential for monitoring the *in vivo* effect of therapeutic agents on autophagy are missing. Until now, no clinical trial has reported to target the core autophagy machinery directly.

In this study, we have undertaken an unbiased approach to evaluate expression of 47 key autophagy genes in a variety of human cancer samples. We find evidence that certain autophagy genes are prevalently altered over others and potential hotspot mutations are found in members of the ULK family of kinases, RB1CC1, PIK3C3 and VCP. We identified a frequent mutation of arginine 70 in MAP1LC3A that has led us to study the functional consequence of this mutation in more detail. We observed reduced LC3A processing and LC3A-positive puncta formation as a consequence of the R70H mutation, suggesting a functional role. In summary, our study proposes that each individual tumour type displays a different autophagic signature that should be taken into account when designing therapeutic approaches.

## RESULTS

### Frequent autophagy gene amplifications in multiple cancers

**Figure 1:**
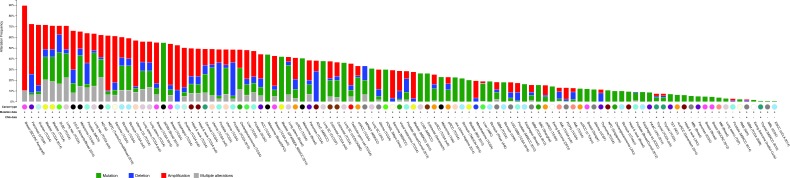
Cumulative frequency of autophagy gene expression changes in various tumour types 47 autophagy genes as listed in Supplemental Table 1 were investigated for copy number alterations in various cancer types that are included in the cBIO portal cancer sequencing database (www.cbioportal.org). Shown is the percentage of copy number alterations in each cancer sub-type. For a full list of abbreviations, please see Supplementary Table 1. Shown in blue colour are copy number deletions, in red are copy number amplifications and in green are mutations.

In order to gain insight into the frequency of copy number alterations and mutational status of autophagy genes in tumour samples, we analyzed a set of 47 key autophagy genes (Supplementary Table 1) in the cBIO cancer genomics portal. These genes include components of the autophagy machinery and proteins involved in lysosomal function, but exclude general signaling molecules that have pleiotropic functions in mitogenic signaling (such as AKT or mTOR). Firstly, we assessed the cumulative frequency of copy number alterations for all 47 genes in a variety of cancers (Figure 1). We found that at least one autophagy gene is altered frequently in multiple cancer types including Pancreatic Cancer (72.5%; 79 cases), Ovarian Serous Cystadenocarcinoma (71.7%; 223 cases), Bladder Urothelial Carcinoma (71.7%; 91 cases), Lung Squamous Cell Carcinoma (70.8%; 126 cases), Skin Cutaneous Melanoma (65.5%; 182 cases) and Stomach Adenocarcinoma (60.3%; 173 cases). Supplementary Table 1 and Supplementary File 1 summarises the frequency of overall alterations (copy number alterations plus mutations) in all cancer studies analysed.

The majority of autophagy gene alterations were copy number amplifications and - at a lower frequency - copy number deletions and mutations in autophagy genes. In contrast, in Prostate Adenocarcinoma a large number of copy number deletions were found, mainly in the ATG5 and ZBTB24 gene (Supplementary File 1).

Interestingly, some cancers show very low copy number alterations. For example, alterations in autophagy genes were much less common in Thyroid Carcinoma (7.5%) and Glioblastoma (2.2%) than in other cancer types. Precise control of autophagy in these types of cancer may provide a benefit to tumour growth and may expose vulnerabilities for anti-cancer strategies.

**Figure 2:**
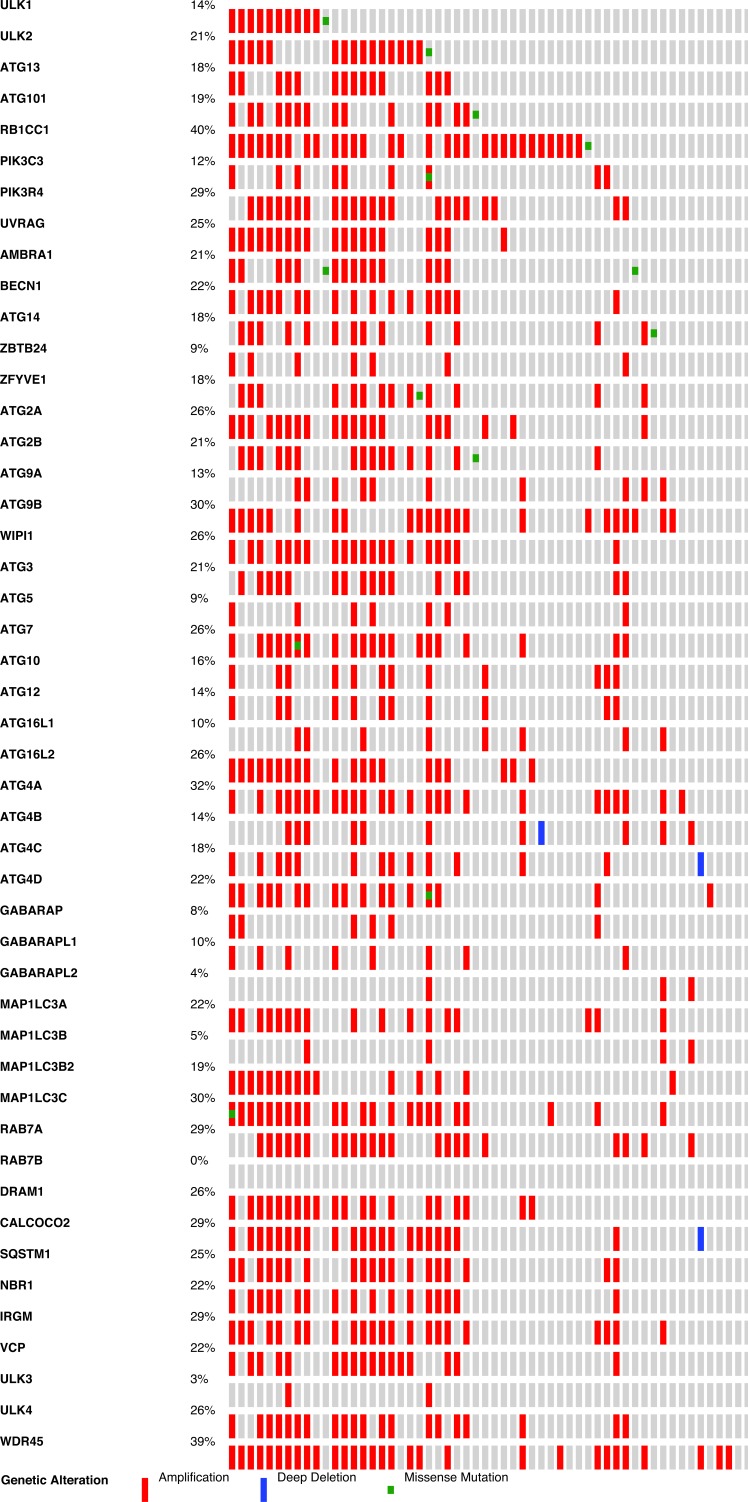
Frequency of autophagy gene expression changes in Neuroendocrine Prostate Cancer Gene alterations for the 47 selected autophagy genes were investigated for copy number amplifications, deletions and mutations in the cBio portal cancer sequencing database. Red colour indicates copy number amplifications and blue colour indicates deletions.

Neuro-endocrine Prostate Cancer displayed an interesting profile, showing gene alterations in 61.7% of cases, with many overlapping amplifications (Figure 2). For example, some cases have copy number amplifications in as many as 34 out of 47 autophagy genes. This remarkable up-regulation of autophagy copy numbers deserves further investigation as it can be expected that the autophagy pathway is highly dysfunctional in this type of cancer.

**Figure 3:**
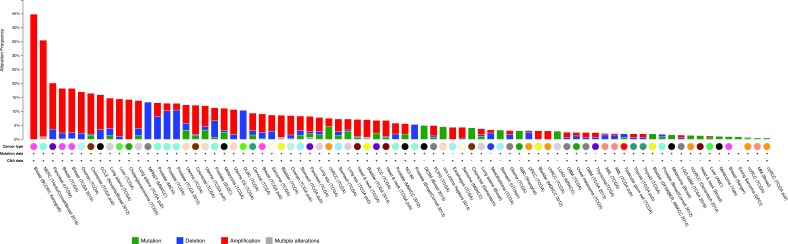
Frequency of LC3 family member gene expression changes in various cancer types The cumulative gene expression changes for 4 LC3 family members are shown (MAP1LC3A, MAP1LC3B, MAP1LC3B2, MAP1LC3C). Shown in blue colour are copy number deletions, in red are copy number amplifications and in green are mutations.

Next, we analyzed individual autophagy gene amplifications present in the studied cancer types (Supplementary File 1 and Supplementary Table 2). We selected all cancer types where data is based on more than 100 cases and where more than 50% autophagy gene alterations were detected in the cumulative study. Overall, when looking at single gene alterations, the incidence was relatively low across all 47 genes. Most individual genes are altered at a frequency of less than 10% and there is no single gene that stands out as a candidate tumour driver across multiple cancer types. Instead, individual cancer types show an increased frequency in selected genes. For instance, in Prostate Adenocarcinoma, frequent copy number deletion for ZBTB24 (13%) and for ATG5 (12%) was observed. A markedly high number of copy number deletions were also observed for the PIK3C3 gene in Pancreatic cancer (24%), whereas copy number amplifications for ATG9B (17%), ATG4B (16%) and MAP1LC3C (13%) were commonly found. MAP1LC3C was also frequently amplified in Breast Cancer (14%) and Ovarian Serous Cystadenocarcinoma (10%). Overall, the LC3 family of proteins together account for 44.8% of gene amplifications in Breast cancer patient xenografts and 35.5% of amplifications in Neuroendocrine Prostate Cancer (Figure 3), suggesting an important role for this gene family in cancer. Most single gene alterations were non-overlapping in the same samples, with exception of Neuroendocrine Prostate Cancer which showed remarkable overlap of multiple autophagy gene amplifications in the same samples.

### Autophagy gene mutations in cancer

In addition to copy number alterations, the frequency of gene sequence mutations is an important feature to assess the role of autophagy genes in cancer. Thus, we investigated the frequency of gene mutations in the 47 selected autophagy genes.

**Figure 4:**
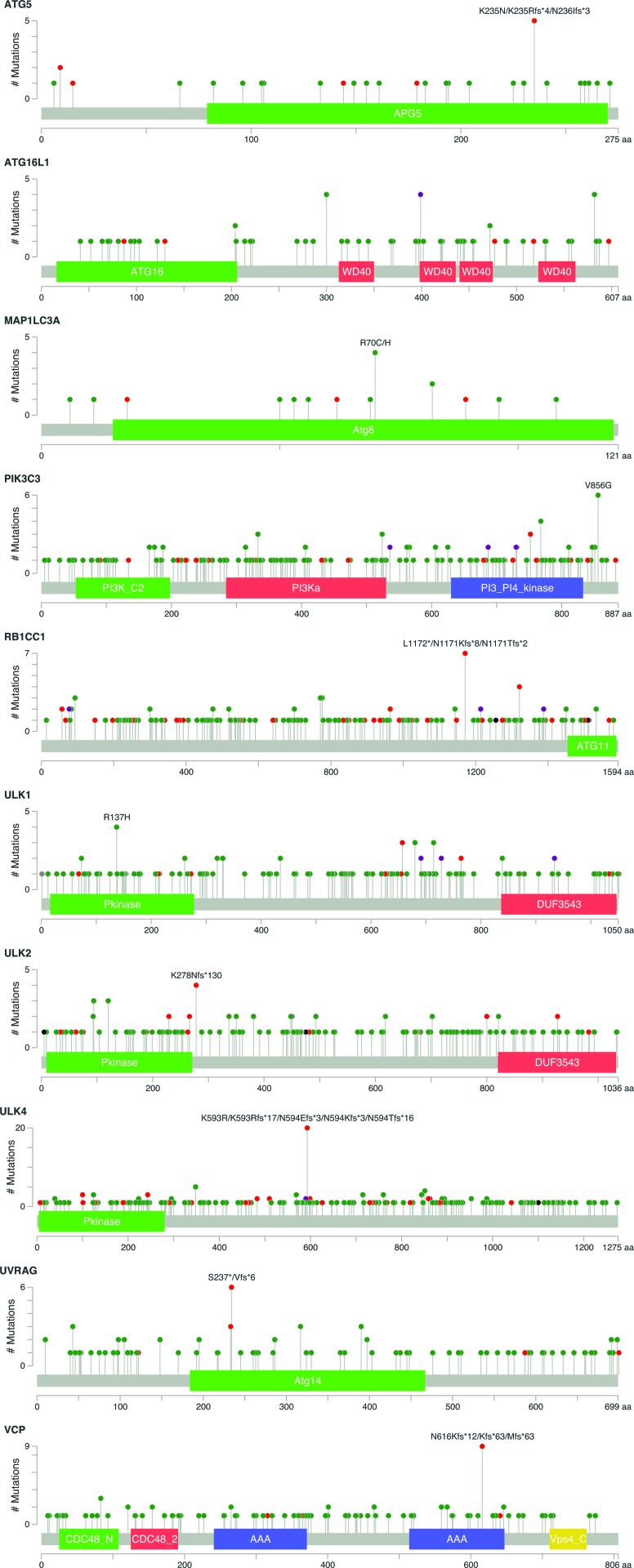
Mutations in selected autophagy genes in various cancer types **A**, The position of frequent gene mutations identified in the cBioportal cancer database for ten autophagy genes are shown. Green colour indicates missense mutations and red colour indicates nonsense or splice mutations.

We were particularly interested to identify genes demonstrating hotspot mutations, which may be indicative of a causal link between cancer development and gene function. Overall, we found ten genes with potential hotspot mutations at one specific site (Figure 4): ATG16L1 (4 cases, T300A), ATG5 (5 cases K235/236 frameshift), MAP1LC3A (4 cases, R70C/H), PIK3C3 (6 cases, V856G), RB1CC1 (7 cases, L1171/1172 frameshift), ULK1 (4 cases, R137H), ULK2 (4 cases, K178 frameshift), ULK4 (20 cases, K593 frameshift), UVRAG (6 cases, S237 frameshift) and VCP (9 cases N616 frameshift). The functional consequence of most of these mutations result in a deficient protein, for instance by premature termination as a consequence of frameshifting (Table 1). However, for some genes the consequence of mutation is less clear and may result in functional protein. The T300A mutation for ATG16L1 has been previously reported as a risk allele for Crohn's disease [18]. The T300A mutation renders the protein more susceptible to caspase-mediated cleavage, thus resulting in reduced stability [19, 20] and intracellular pathogen clearance [21]. It is interesting to note that the same mutation occurs in some cancer samples and so reduced activity of ATG16L1 may have functional consequences in other diseases as well as Crohn's disease.

**Table 1:** Hotspot mutations in human autophagy genes in cancer samples

Gene	Mutation	Cancer Type	Functional consequence
ATG16L1	T300A R399*/L/P A582V/E	Diffuse Large B-Cell Lymphoma Multiple Multiple	Reduced stability/loss Missense/nonsense Missense
ATG5	K235/236fs	Stomach Adenocarcinoma	FS deletion
MAP1LC3A	R70H	Stomach/Pancreas	Missense
PIK3C3	V856G	Invasive Breast Carcinoma	Missense
RB1CC1	L1171/1172fs	Stomach/SCLC	FS insertion/deletion
ULK1	R137H	Multiple	Missense
ULK2	K178fs	Stomach Adenocarcinoma	FS deletion
ULK4	K593fs	Multiple	FS insertion/deletion
UVRAG	S237fs	Multiple	FS deletion
VCP	N616fs	Multiple	FS insertion/deletion

Abbreviations: fs – frameshift, SCLC – small cell lung cancer.

**Figure 5:**
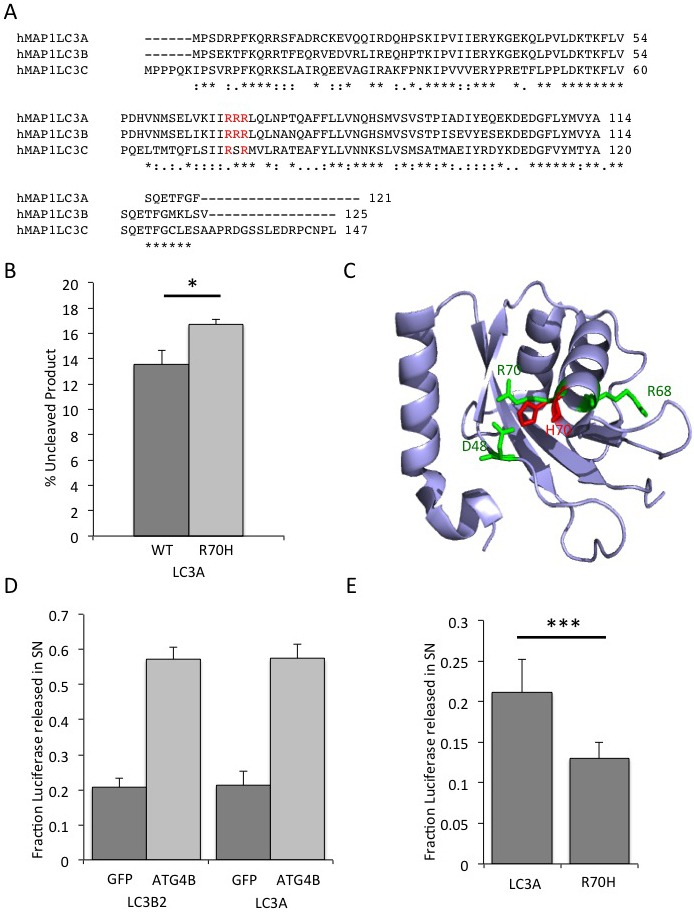
Reduced processing of LC3A R70H *in vitro* **A.**, sequence alignment of human MAP1LC3A, MAP1LC3B and MAP1LC3C. Alignment was done using ClustalW. **B.**, ATG4B-mediated cleavage of MAP1LC3A R70H-GST is slower than cleavage of MAP1LC3A wild-type. Recombinant MAP1LC3A-GST and MAP1LC3A R70H-GST were incubated with ATG4B for 20 min at 37°C and resolved by polyacryalamide gel electrophoresis. The bands for uncleaved and cleaved MAP1LC3A-GST were quantified using IMAGEJ and normalized to uncleaved MAP1LC3A-GST at time 0. **C.**, Structure of MAP1LC3A (PDB: 3WAL). R68 and R70 are highlighted in green. Amino acid side chains of Asp48, Arg68, Arg70 and mutated R70H are shown as sticks in green and red. Salt bridges are shown in dashed blue lines. The Figure was generated using PyMOL. **D.**, LC3A processing in response to ATG4B. The Actin-LC3B2-dNGLUC and Actin-LC3A-dNGLUC luciferase release reporter were co-transfected with GFP as control or ATG4B in HEK293 cells by lipofection. Supernatants were harvested 48h thereafter and cells were lysed in lysis buffer. The total amount of luciferase was calculated in supernatants and cell lysates. Released luciferase as a percentage of total luciferase is an indicator of processing of both LC3 homologues. **E.**, Luciferase release of Actin-LC3A R70H-dNGLUC is reduced compared to wild-type Actin-LC3A-dNGLUC. The fraction of released luciferase was calculated as in Figure 5D.

Another gene mutation with potential functional consequence is the MAP1LC3A R70H substitution. Previously, a functional role for residue R68 and surrounding residues in MAP1LC3A has been proposed [22]. Thus, mutation of this residue may result in a change of conformation that can affect the function of LC3 and result in reduced autophagic flux. The arginine-rich motif R68-R70 is highly conserved in other LC3 members including MAP1LC3B and MAP1LC3C (Figure 5A). We next explored whether mutations in this region in MAP1LC3 family members were more commonly found in other cancer databases as well. Overall, we found 3 R69H and 6 R70C/H mutations in MAP1LC3A and 4 R76C/H mutations in MAP1LC3C in the cBio, ICGC and COSMIC databases (Table 2).

**Table 2:** Mutations within the arginine-rich stretch (R68-70) in MAP1LC3A and corresponding sequence in MAP1LC3C

Gene	Mutation	Cancer Type	Sample ID	Database
hMAP1LC3A	R69H	Cervix Carcinoma	TCGA-DR-A0ZM	COSMIC [COSM459773] and cBio Portal
	R69H	Pancreas	8066440	COSMIC [COSM459773]
	R69H	Pancreas	DO49141	ICGC [PACA-AU]
	R70C	Upper Aerodigestive tract	HN_62854	COSMIC (COSM124308] and cBio Portal
	R70H	Stomach	DO38567	ICGC [STAD-US]
	R70H	Stomach	DO48032	ICGC [STAD-US]
	R70H	Stomach	TCGA-BR-8361	cBio Portal
	R70H	Stomach	TCGA-CG-4306	cBio Portal
	R70H	Pancreas	TCGA-IB-7651	cBio Portal
hMAP1LC3C	R76C	Endometrium	TCGA-AX-A0J1	COSMIC [COSM906534] and cBio Portal
	R76C	Large Intestine	TCGA-AZ-6598	COSMIC [COSM906534]
	R76H	Kidney	TCGA-BP-5202	COSMIC [COSM1492022]
	R76R	Stomach	DO10276	ICGC [COAD-US]
	R76H	Melanoma	MEL-JWCI-WGS-34	cBio Portal

Only separate sample donors identified in the three databases COSMIC, ICGC and cBio Portal are shown for the respective mutations. Overall, 14 somatic mutations in the triple arginine-stretch of MAP1LC3A and the corresponding sequence in MAP1LC3C were found.

### Slower processing of MAP1LC3A R70H compared to wild-type MAP1LC3A

All LC3 family members undergo multiple processing steps to convert a pro-LC3 form into the autophagosome-associated LC3-II form that bears a lipid anchor at the C-terminus. This proteolytic processing is mediated by autophagy proteases of the ATG4 family member *via* an intermediate LC3-I form that has a C-terminal truncation at G120 and sub-sequent addition of a lipid anchor by ATG3/7. In order to investigate whether the R70H mutation results in altered MAP1LC3A cleavage, we tested ATG4B-mediated processing of MAP1LC3A using an *in vitro* cleavage assay. Briefly, MAP1LC3A tagged with GST at the C-terminus was co-incubated with recombinant ATG4B and cleavage activity was determined by quantification of the cleaved bands visualized on a protein gel over time (Supplementary File 2). Overall, MAP1LC3A R70H displayed a slower kinetic for ATG4B-mediated cleavage (Figure 5B), pointing to a functional role of the R70 residue in this context. *In silico* modeling of the R70H substitution using a crystal structure of MAP1LC3A (PDB:3WAL) shows a conformational change that leads to re-arrangement and co-ordination of the imidazole ring of R70H adjacent to residue D48 (Figure 5C). Overall, the substitution of R70 by a histidine may result in a structural conformation change that is less favourable for cleavage.

In order to investigate the cleavage of MAP1LC3A and the R70H mutant in cells, we generated a cell-based LC3A cleavage reporter assay based on the luciferase release assay that was previously described for MAP1LC3B2 [23]. This reporter is based on release of *Gaussia luciferase* from an Actin-LC3 tether into supernatants by unconventional secretion [24]. The amount of luciferase released into supernatants correlates with ATG4B-mediated cleavage of the reporter. First, we tested the functionality of the Actin-MAP1LC3A-dNGLUC reporter by assessing the response to ATG4B overexpression. As shown in Figure 5D, basal levels of luciferase release were similar for the LC3B2 and LC3A reporter (20.7% and 21.2%, respectively). Upon transfection of ATG4B, 57.2% and 57.4% of total luciferase activity was found in supernatants for LC3B2 and LC3A, respectively. These results demonstrate that the Actin-LC3A-dNGLUC reporter is functional and LC3A is a cleavable substrate for ATG4B. Next, we compared the release of luciferase for MAP1LC3A and MAP1LC3A R70H. Basal levels of release were significantly reduced for the R70H mutant, displaying only 12.9% of total luciferase activity in supernatants when compared to wild-type LC3A with 21.2% of total luciferase activity in supernatants (Figure 5E). These results confirm the *in vitro* cleavage data demonstrating that LC3A R70H has a slower processing when compared to wild-type LC3A.

**Figure 6:**
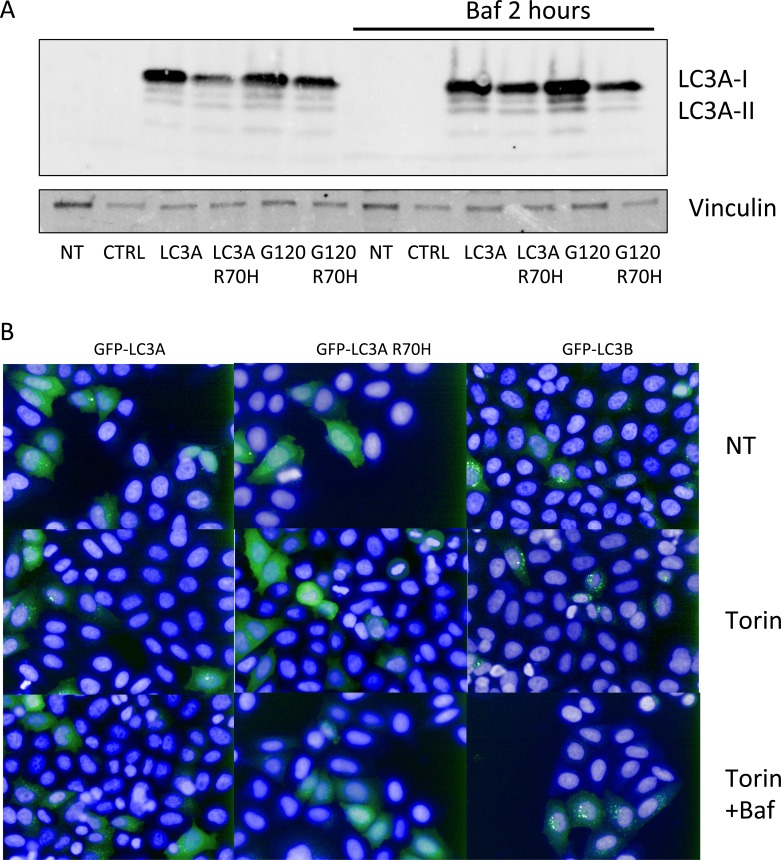
Reduced LC3A R70H processing in cells **A**. Flag-tagged LC3A and LC3A R70H were transfected in HEK293T cells and left untreated or treated with Bafilomycin A. Cell lysates were resolved by SDS-PAGE and analysed by western-blotting using anti-Flag antibodies. The R70H mutant displays a reduction in LC3A-II that does not accumulate upon treatment with Bafilomycin A1. Vinculin was used as loading control. NT - not transfected; CTRL - transfected with empty control vector. **B.**, 293T cells were transfected with GFP-tagged LC3B, LC3A and LC3A R70H and subsequently treated with Torin 1 (250nM) or Torin1 plus Bafilomycin A and compared to DMSO treated cells. Cells were counter-stained with Hoechst 33342 to identify nuclei.

In order to confirm a reduced capacity for LC3A processing in cells, we transfected Flag-tagged LC3A and LC3A R70H mutant in HEK293 cells and investigated the abundance of LC3A-I and LC3A-II forms by western-blotting. The major form in untreated cells is LC3A-I (upper band in the blot shown in Figure 6A) with two lower bands that correspond to lipidated LC3-II. We noticed that LC3A R70H is expressed at significantly lower levels than LC3A, but shows the same pattern with higher levels of LC3A-I over LC3A-II (Figure 6A). Upon treatment with Bafilomycin A, the levels of LC3A-II increased for LC3A but not for LC3A R70H, suggesting that autophagic flux is reduced for the R70H mutant (Figure 6A). This effect is even more pronounced for LC3A^G120^ and LC3A^G120^ R70H, C-terminal truncations that by-passes pro-LC3A cleavage. When cells are expressing LC3A^G120^ R70H, the levels of LC3A R70H-II were significantly reduced when compared to the WT LC3A^G120^-II, indicating that the majority of the R70H isoform is not lipidated (Figure 6A). In summary, these results indicate that processing of LC3A R70H is reduced compared to WT LC3A, both *in vitro* and *in vivo*.

Finally, we were interested to see if reduced processing of LC3A R70H manifests as a reduction in LC3A-positive autophagosomes. We therefore expressed GFP-LC3A and GFP-LC3A R70H in HEK293 cells and monitored GFP-positive puncta by fluorescence microscopy (Figure 6B). Generally, we observed a lower number of LC3A-positive puncta than LC3B-puncta. Upon treatment with Torin, a compound that enhances autophagy, the number of both LC3A- and LC3B-positive puncta increases, an effect that can be further enhanced by co-treatment of cells with Torin and Bafilomycin A (Figure 6B). In contrast, cells expressing GFP-LC3A R70H did not show any appearance of LC3A R70H-positive puncta even when cells were treated with Torin plus Bafilomycin A. These results indicate that LC3A R70H does not partition into autophagosomal puncta, most likely due to a processing defect.

## DISCUSSION

The importance of autophagy in tumour initiation and maintenance has been recognized but efforts to identify inhibitors of autophagy core proteins for anti-cancer strategies have failed so far [25]. In addition, therapeutic development has been hampered by the complexity and contextual contradictory of autophagy, especially in the context of cancer [26]. The prevailing view is that autophagy may present a barrier in the initial stages of transformation, thus supporting the function as a tumour suppressor, whereas once this barrier is overcome, autophagy may benefit continued tumour growth at later stages [4]. Our results reflect a static snapshot of autophagy gene expression in various cancers and do not take into account the developmental or dynamic nature of individual cancer types. However, this analysis provides important findings underscoring the relevance of autophagy genes in cancer biology.

In this study, we have explored two areas: firstly, we analysed autophagy gene alterations and mutations in various cancer types as extracted from publicly available data sets, and secondly, we interrogated whether individual autophagy gene products are significantly altered in various cancers. We have taken an unbiased and systematic approach to gain an overview of gene expression patterns of autophagy genes in a wide variety of human tumour samples. Overall, we observed that autophagy gene amplifications were more commonly observed than gene deletions in most cancer types. In contrast, in other tumour types such as thyroid cancer and glioblastoma, autophagy gene alterations were not present even though the datasets consisted of large numbers. As a consequence, these cancer types may be particularly vulnerable to alterations or mutations in autophagy genes as literature supports. There has been good evidence that these cancer patients may therapeutically benefit from enhancing autophagy by treatment with rapamycin or temozolomide [27, 28]. In addition, sensitizing glioblastoma cells by inhibition of autophagy by RNAi renders them more sensitive to apoptosis inducing compounds [29]. These findings underscore that glioblastoma offers particular vulnerabilities for therapeutic targeting in the autophagy pathway.

Alternatively, specific cancer driver mutations may exhibit a linkage to specific sets of altered autophagy genes in the context of cancer progression. This aspect was not addressed in our study, and accordingly, we cannot make any prediction whether alterations in any of the autophagy genes are causal to specific cancer development and progression. Also, the contribution of individual autophagy gene expression needs to be addressed experimentally, e.g. by systematic knockout, knock-in or genome editing of each autophagy gene or combinations across a variety of *in vitro* and *in vivo* models.

Another study recently reported cross-cancer profiling on a larger set of genes, including signaling molecules or potential interaction partners of the core proteins, using data available from The Cancer Genome Atlas (TCGA) [30]. A total of 211 genes was analysed in 11 cancer types, including invasive breast carcinoma, glioblastoma multiforme, acute myeloid leukemia and ovarian cystadenocarcinoma. A very low frequency of mutations in individual autophagy core genes in these cancer types was reported, which is in agreement with our study. The authors suggested that the autophagy machinery may be protected from alterations in some human cancers, supporting the notion that autophagy may be essential for progression of some tumour types. We largely agree with this assessment, and hypothesize that drug targeting of the autophagy core machinery may expose vulnerabilities for anti-cancer strategies.

In this study we found a common mutation hotspot in four autophagy proteins occurring at a higher than average frequency in various cancers. One of them, MAP1LC3A is a member of the LC3 family of proteins and a key structural component of the autophagosome that undergoes processing by members of the ATG4 family, most notably ATG4B. LC3 is first proteolytically cleaved to generate a C-terminal truncation, LC3-I that is then conjugated to phosphatidyl-ethanolamine to generate LC3-II. In the later stages of autophagy, LC3-II is de-conjugated by ATG4 to recycle LC3-I. The kinetics of LC3 processing is generally viewed as an indicator of autophagy flux. The identified MAP1LC3A R70C/H mutation substitutes a critical residue in MAP1LC3A that is required for LC3A processing. In the absence of ATG4, R70 and D48 form salt bridges (Figure 5C) while the side chain of R68 points to the interface of the protein. In complex with ATG4B, the salt bridges between R70 and D48 break down and R68 forms new salt bridges with D171 of ATG4B (see crystal structure PDB:2Z0E). On LC3 binding, the regulatory loop and the N-terminal tail of ATG4B undergo large conformational changes. The open conformation could be stabilized by the interaction with non-substrate LC3, specifically forming salt bridges between R70 of LC3A and D9 of ATG4B, and favour membrane targeting of ATG4B that is required for LC3-PE de-conjugation [31]. Thus, mutations in the arginine-rich stretch of LC3 result in structural re-arrangements that can account for decreased processing by ATG4 family members. This is underscored by findings that the surrounding residue R68 is deleted in a non-functional splice isoform of MAP1LC3A [22]. R68 is involved in binding of ATG4B and a R68A mutant has been shown to result in reduced cleavage by ATG4B [31]. This region has been implicated in cardiolipin binding and elimination of damaged mitochondria by mitophagy [32]. Overall, mutations in this stretch of residues R68, R69, R70 account for 9 cases of MAP1LC3A mutations and R76 mutations for 5 cases of MAP1LC3C in cancer. We suggest that slower processing of LC3A can result in a reduced autophagic flux and reduced availability of LC3-I for responses to cellular stress or damage. An alternative hypothesis is that R70 contributes to the binding of LIR domain proteins that then affect autophagosome maturation. For instance, it has recently been observed that FYCO-1, a protein required for autophagosome transport along microtubules [33] binds directly *via* its LIR domain to a region in LC3A and LC3B that includes R70, thereby affecting autophagosome maturation under basal conditions [34]. The precise mechanisms how a R70H substitution contributes to defective autophagosome maturation will need to be studied in further detail.

Recently, a distinct role for LC3A in cancer cell lines has been described [35]. The authors demonstrate that LC3A-positive autophagosomes are distinct from LC3B-positive autophagosomes and cell- or tissue-specific roles for LC3A as opposed to LC3B may exist. LC3A has been identified as a biomarker in multiple cancer types including colorectal adenocarcinoma [36], non-small cell lung carcinoma [37], cutaneous malignant melanomas [38] and breast carcinoma [39]. Furthermore, it has been proposed that LC3A is frequently inactivated in human cancers [40], thus corroborating our observation that reports a defective LC3A variant in human cancer samples.

The conclusions based on the reported analysis may be crucial for designing therapeutic strategies based on the use of autophagy inhibitors and activators and will have implications for the development of autophagy modulators as anti-cancer therapeutic agents. In particular, the role of defective LC3A processing in cancer warrants further investigation and a more detailed study into the role of LC3 family members in autophagosome formation and maturation in cells and tissues.

## MATERIALS AND METHODS

### Cancer gene expression database

The cBIO Cancer Genomics Portal (http://cbioportal.org/) is an open access resource for cancer genomics data sets [41] and contains data from 69 cancer genomics studies with a total number of 17,177 samples. A table with references, the number of available samples per cancer study and data type can be found in Supplementary Table 1. 47 autophagy genes were selected for analysis on the basis that their primary function was involved in autophagy regulation rather than other processes. For this reason, signaling genes such as mTOR or AKT were excluded as (1) they are involved in general signal transduction and (2) they are highly relevant for cancer initiation, progression and maintenance and thus would skew the analysis.

### Plasmid cloning

cDNA encoding human ATG4B full length was obtained from pEAK-ATG4B [42] by digestion with *EcoR*I and *Not*I restriction enzymes and inserted into bacterial expression vector pGEX6P-1 (GE Healthcare, 27-4597-01) in frame with GST-tag at the N-terminal creating pGEXATG4B. cDNA encoding MAP1LC3A was obtained by PCR from the ORFeome library (ThermoFisher) as templates and using primers LC3A_NcoI and LC3A_R (Supplementary File 1 and inserted to pET21-SmaI-GST in frame at *Nco*I and *Sma*I restriction sites creating pETLC3A-GST. pET21-SmaI-GST was constructed by digestion of pET21d (Novagen, 69743-3) with *BamH*I and restriction digest with *Hind*III. SmaI-GST was obtained by PCR using pGEX6P-1 as template and primers SmaI-GST and GST_HIndIII_R. The DNA was phosphorylated and digested with *Hind*III and inserted into pET21. pETLC3A70H-GST (for mutation of LC3A R70 to H70) was created by PCR using pETLC3A-GST as a template and primers LC3AR70H_F and LC3AR70H_R. All PCR were done at 30 cycles using Pyrobest DNA polymerase (Takara, R005A). Triple (3x) flag-LC3A, -LC3A R70H, -LC3A^G120^ and -LC3A^G120^ R70H were generated by sub-cloning the fragments (originally cloned into pDONR233) into a 3x flag-N terminus destination vector using the Gateway Recombination System (Invitrogen), according to the manufacturer's protocol. Plasmids were verified by sanger sequencing.

The luciferase release reporter Actin-LC3A-dNGLUC and Actin-LC3A R70H-dNGLUC were generated by replacing LC3B2 sequence in the original luciferase release reporter [42] with the corresponding LC3A sequence. MAP1LC3A-dNGLUC and MAP1LC3A R70H-dNGLUC were synthesized by Genscript and inserted into pEAK12-Actin-LC3B2-dNGLUC by restriction cloning using *EcoR*I and *Not*I. pEAK12-GFP-LC3A and pEAK12-GFP-LC3A R70H were generated by removing dNGLUC from the constructs described above, using *NheI* and *NotI*, followed by 30 minute incubation with Klenow large fragment DNA Polymerase (NEB).

### Cell culture and transfection

293T cells were grown in Dulbecco's modified Eagle's medium containing 10% fetal bovine serum, sodium pyruvate and penicillin/streptomycin. Cells were seeded at the density of 1×10^5^ cells/well (12 well plate) or 7.5×10^3^ cells/well (96 well plate) and transfected on the following day with 1ug or 250 ng of plasmid, respectively, for 48 hours, using X-tremeGENE (Roche) or Lipofectamine 2000 (Invitrogen) and according to the manufacturer's protocol. Before harvesting, medium was replaced and cells were treated for 2 hours with Bafilomycin A1 (10 nM).

### Expression and purification

Expression plasmids pGEXATG4B, pETLC3A-GST and pETLC3A70H-GST were transformed into *E. coli* BL21 (DE3)-R3-lambda-PPase obtained from the Structural Genomics Consortium, Oxford University. Protein inductions and purifications were conducted as described before for CLK2cd [43]. GST-ATG4B were digested with PreScission Protease (GE Healthcare, 27-0843-01) and stored at 6 mg/ml at −80°C in 50 mM Tris-HCl, pH 8.0, 150 mM NaCl, 0.5 mM EDTA, 0.1 mM EGTA, 33% glycerol and 1 mM DTT.

### *In vitro* ATG4B proteolysis assay

The assay was done at 37°C in a reaction volume of 20 μl containing 0.00133 μg/μl of ATG4B and 0.525 μg/μl of LC3A-GST or LC3A70H-GST in buffer A containing 150 mM NaCl, 50 mM Tris-HCl pH 8.0 and 2 mM DTT. The reaction was stopped by addition of the same volume of 2X SDS sample buffer and boiling for 5 min. The samples were analysed on a 4-20% Mini-PROTEAN^®^ TGX™ Precast Gel (Bio-Rad, 456-1096) and Coomassie Brilliant Blue Staining. Percentage of the substrate that remain at each reaction time point was equal to OD_LC3-GST_/(OD_LC3-GST_ + OD_GST_ + OD_LC3-I_) × 100%.

### Luciferase release assay

HEK293 cells were transfected using Lipofectamine 2000 according to the manufacturer's instructions. 48 h after transfection of Actin-LC3B2-dNGLUC, Actin-LC3A-dNGLUC and Actin-LC3A R70H-dNGLUC in the presence or absence of ATG4B or GFP as control, supernatants were harvested and cells were lysed in lysis buffer (50 mM Tris HCl, pH 7.5, 150 mM NaCl, 1% NP-40 and protease inhibitors). Luciferase activity was measured using native coelenterazine (Cambridge Bioscience) in *Gaussia luciferase* analysis buffer [24]. In order to account for different transfection efficiencies, the amount of released luciferase as a percentage of total luciferase in both supernatant and lysate was calculated.

### Western-blotting

Cells were collected, washed with 1 ml PBS and resuspended in 100 μl of cold lysis buffer containing. Lysate was cleared by centrifugation at 14,000 rpm for 15 minutes. 10 μg of lysate was mixed with 5x sample buffer and boiled for 5 minutes. The proteins were resolved by SDS-PAGE (4-20%) and transferred to a PVDF membrane. After blocking with 5% milk PBS/tween for 1 h, the membrane was probed with an anti-Flag antibody (M2, Sigma) or anti-Vinculin (ab18058, Abcam) and then washed and exposed to anti-secondary immunofluorescence antibodies (Li-cor Biosciences). Detection was performed on the Odyssey Imaging System (Li-cor Biosciences).

### Fluorescence microscopy and image acquisition

293T cells were transfected with GFP-tagged versions of LC3B, LC3A and LC3A R70H and treated with Torin 1 or Torin 1 plus Bafilomycin A1 for 2h. Cells were then fixed with 4% paraformaldehyde for 15 minutes, washed three times with PBS and stained with Hoechst 33342 (1:10.000). Images were acquired using the Opera High Content Screening System (Perkin Elmer) with a 40x objective in at least 6 replicates per condition. Nuclei were identified on the 365nm channel and GFP-LC3 spots on the 488nm channel the Opera High Content Screening System (Perkin Elmer), using the 488nm and 365nm lasers and objective lens 40X.

## SUPPLEMENTARY MATERIALS FIGURES AND TABLES






