# Supplementary material for: Autophagy gene expression profiling identifies a defective microtubule-associated protein light chain 3A mutant in cancer
Source: Oncotarget. 2016 May 31;7(27):41203–16. doi: 10.18632/oncotarget.9754 (PMC5173052; doi:10.18632/oncotarget.9754)
Supplement: Supplementary file 1 [file oncotarget-07-41203-s001.pdf]

Autophagy gene expression profiling identifies a defective microtubule-associated protein light chain 3A mutant in cancer

Supplementary Material

Supplementary File 1

Gene Alterations

Page 2 – Pancreatic Cancer (UTSW, Nat Commun 2015)
Page 3 – Ovarian Serous Cystadenocarcinoma (TCGA, Provisional)
Page 4 – Bladder Urothelial Carcinoma (TCGA, Nature 2014)
Page 5 – Lung Squamous Cell Carcinoma (TCGA, Provisional)
Page 6 – Skin Cutaneous Melanoma (TCGA, Provisional)
Page 7 – Neuroendocrine Prostate Cancer (Trento/Cornell/Broad 2016)
Page 8 – Sarcoma (TCGA, Provisional)
Page 9 – Stomach (TCGA, Provisional)
Page 10 – Lung Adenocarcinoma (TCGA, Nature 2014)
Page 11 – Prostate Adenocarcinoma, Metastatic (TCGA, Cell 2015)

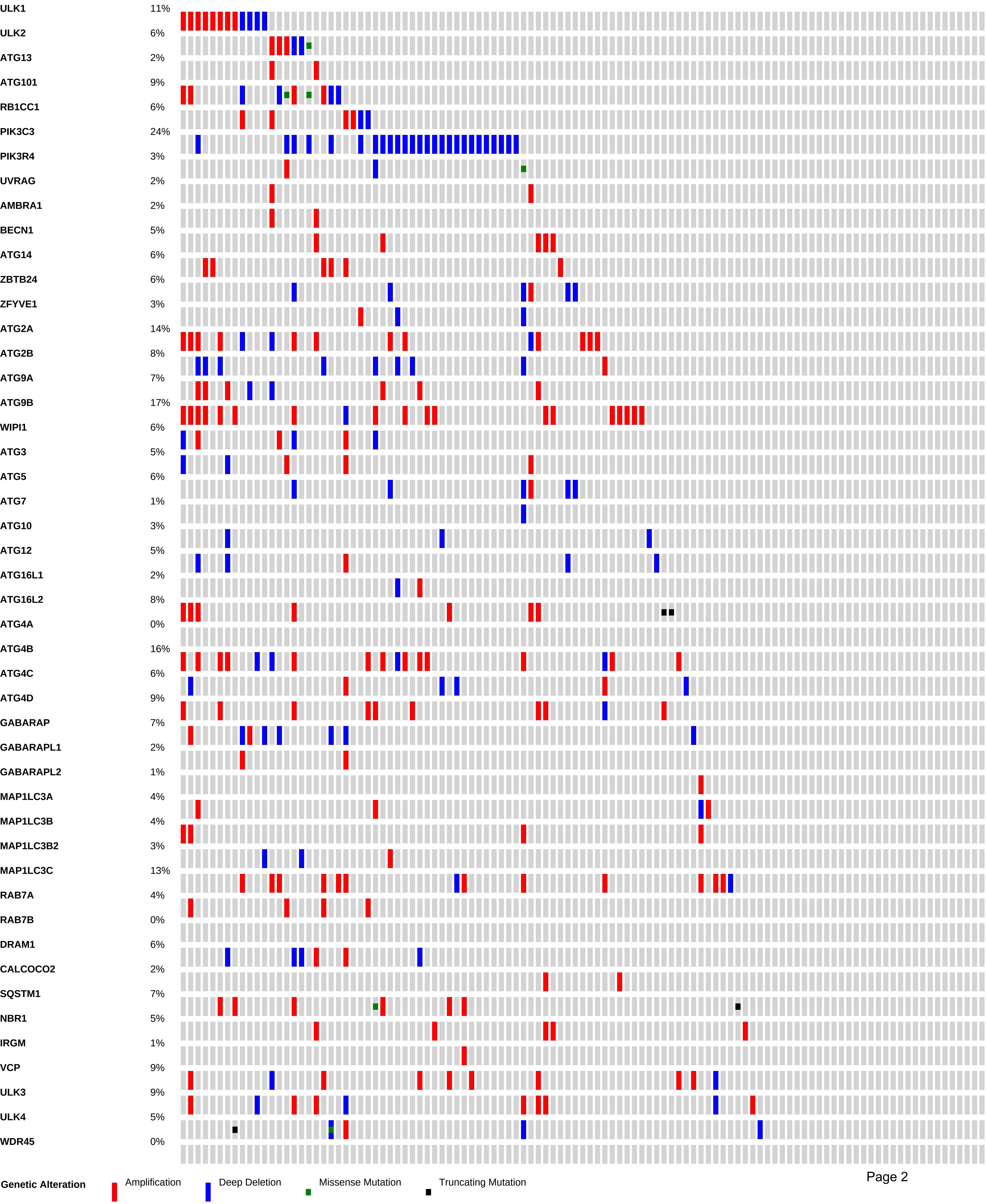
ULK1 11%
ULK2 6%
ATG13 2%
ATG101 9%
RB1CC1 6%
PIK3C3 24%
PIK3R4 3%
UVRAG 2%
AMBRA1 2%
BECN1 5%
ATG14 6%
ZBTB24 6%
ZFYVE1 3%
ATG2A 14%
ATG2B 8%
ATG9A 7%
ATG9B 17%
WIPI1 6%
ATG3 5%
ATG5 6%
ATG7 1%
ATG10 3%
ATG12 5%
ATG16L1 2%
ATG16L2 8%
ATG4A 0%
ATG4B 16%
ATG4C 6%
ATG4D 9%
GABARAP 7%
GABARAPL1 2%
GABARAPL2 1%
MAP1LC3A 4%
MAP1LC3B 4%
MAP1LC3B2 3%
MAP1LC3C 13%
RAB7A 4%
RAB7B 0%
DRAM1 6%
CALCOCO2 2%
SQSTM1 7%
NBR1 5%
IRGM 1%
VCP 9%
ULK3 9%
ULK4 5%
WDR45 0%
Genetic Alteration
Amplification
Deep Deletion
Missense Mutation
Truncating Mutation

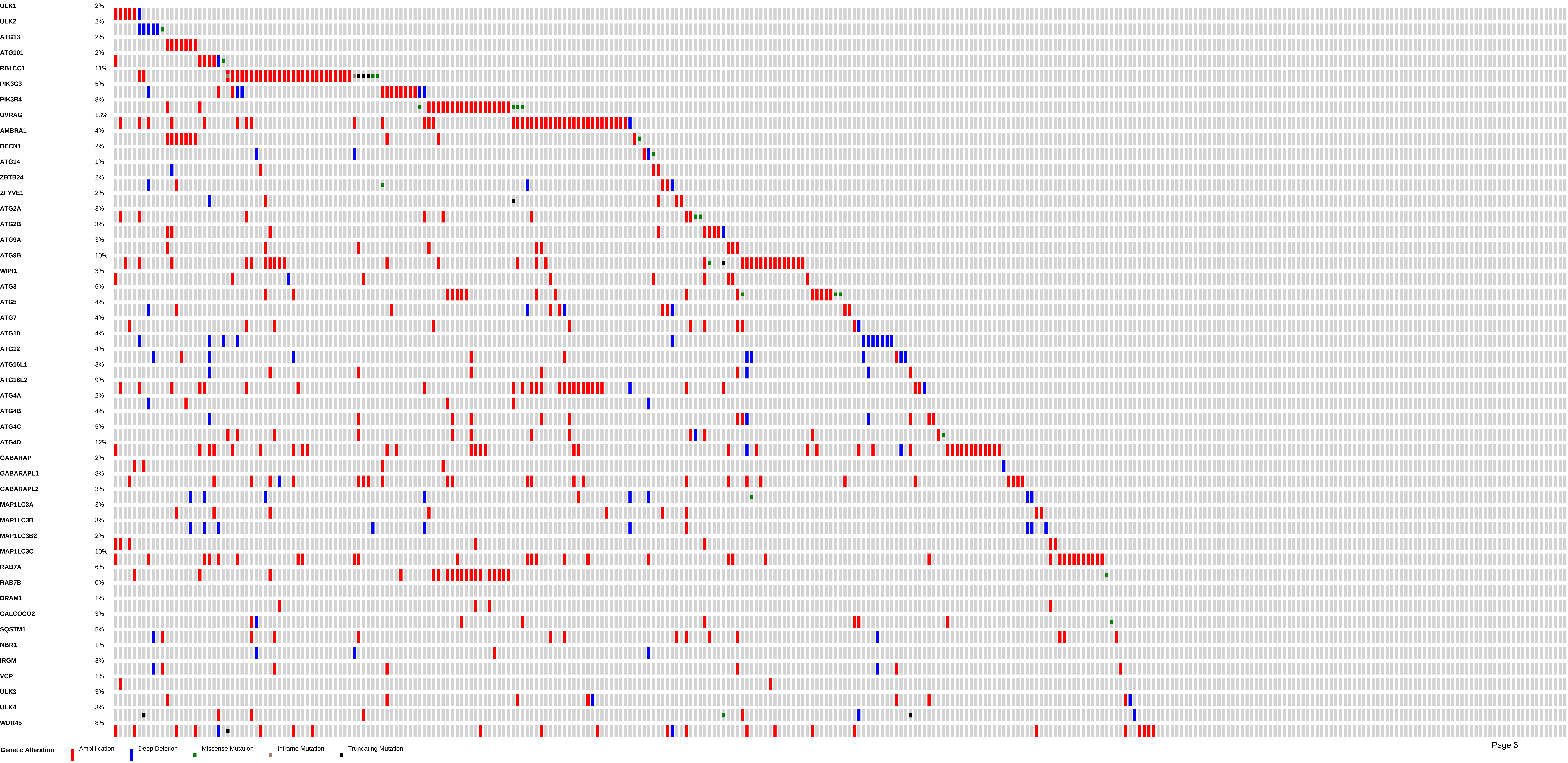
ULK1 2%
ULK2 2%
ATG13 2%
ATG101 2%
RB1CC1 11%
PIK3C3 5%
PIK3R4 8%
UVRAG 13%
AMBRA1 4%
BECN1 2%
ATG14 1%
ZBTB24 2%
ZFYVE1 2%
ATG2A 3%
ATG2B 3%
ATG9A 3%
ATG9B 10%
WIPI1 3%
ATG3 6%
ATG5 4%
ATG7 4%
ATG10 4%
ATG12 4%
ATG16L1 3%
ATG16L2 9%
ATG4A 2%
ATG4B 4%
ATG4C 5%
ATG4D 12%
GABARAP 2%
GABARAPL1 8%
GABARAPL2 3%
MAP1LC3A 3%
MAP1LC3B 3%
MAP1LC3B2 2%
MAP1LC3C 10%
RAB7A 6%
RAB7B 0%
DRAM1 1%
CALCOCO2 3%
SQSTM1 5%
NBR1 1%
IRGM 3%
VCP 1%
ULK3 3%
ULK4 3%
WDR45 8%
Genetic Alteration
Amplification
Deep Deletion
Missense Mutation
Inframe Mutation
Truncating Mutation

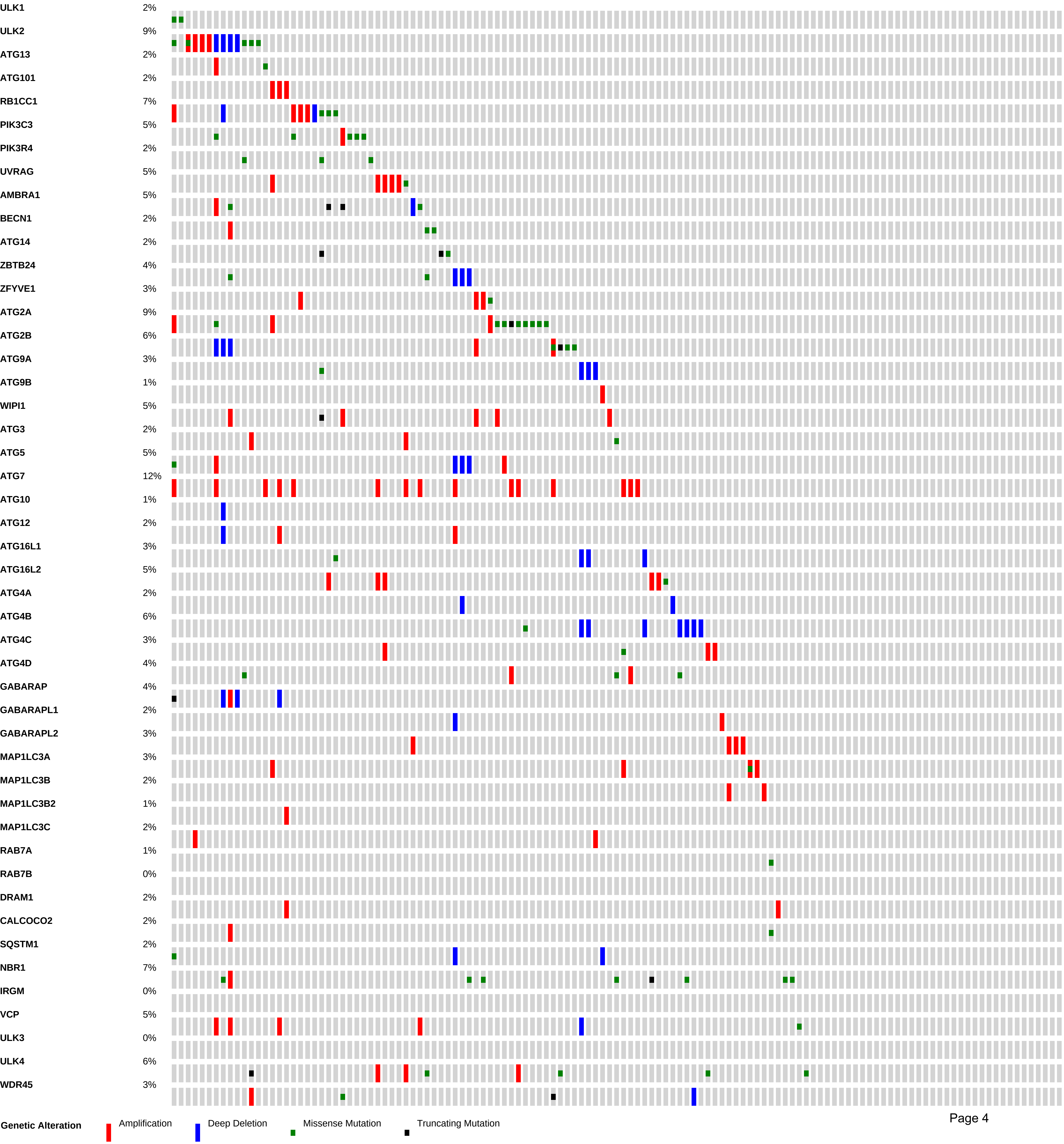
ULK1 2%
ULK2 9%
ATG13 2%
ATG101 2%
RB1CC1 7%
PIK3C3 5%
PIK3R4 2%
UVRAG 5%
AMBRA1 5%
BECN1 2%
ATG14 2%
ZBTB24 4%
ZFYVE1 3%
ATG2A 9%
ATG2B 6%
ATG9A 3%
ATG9B 1%
WIPI1 5%
ATG3 2%
ATG5 5%
ATG7 12%
ATG10 1%
ATG12 2%
ATG16L1 3%
ATG16L2 5%
ATG4A 2%
ATG4B 6%
ATG4C 3%
ATG4D 4%
GABARAP 4%
GABARAPL1 2%
GABARAPL2 3%
MAP1LC3A 3%
MAP1LC3B 2%
MAP1LC3B2 1%
MAP1LC3C 2%
RAB7A 1%
RAB7B 0%
DRAM1 2%
CALCOCO2 2%
SQSTM1 2%
NBR1 7%
IRGM 0%
VCP 5%
ULK3 0%
ULK4 6%
WDR45 3%
Genetic Alteration
Amplification
Deep Deletion
Missense Mutation
Truncating Mutation

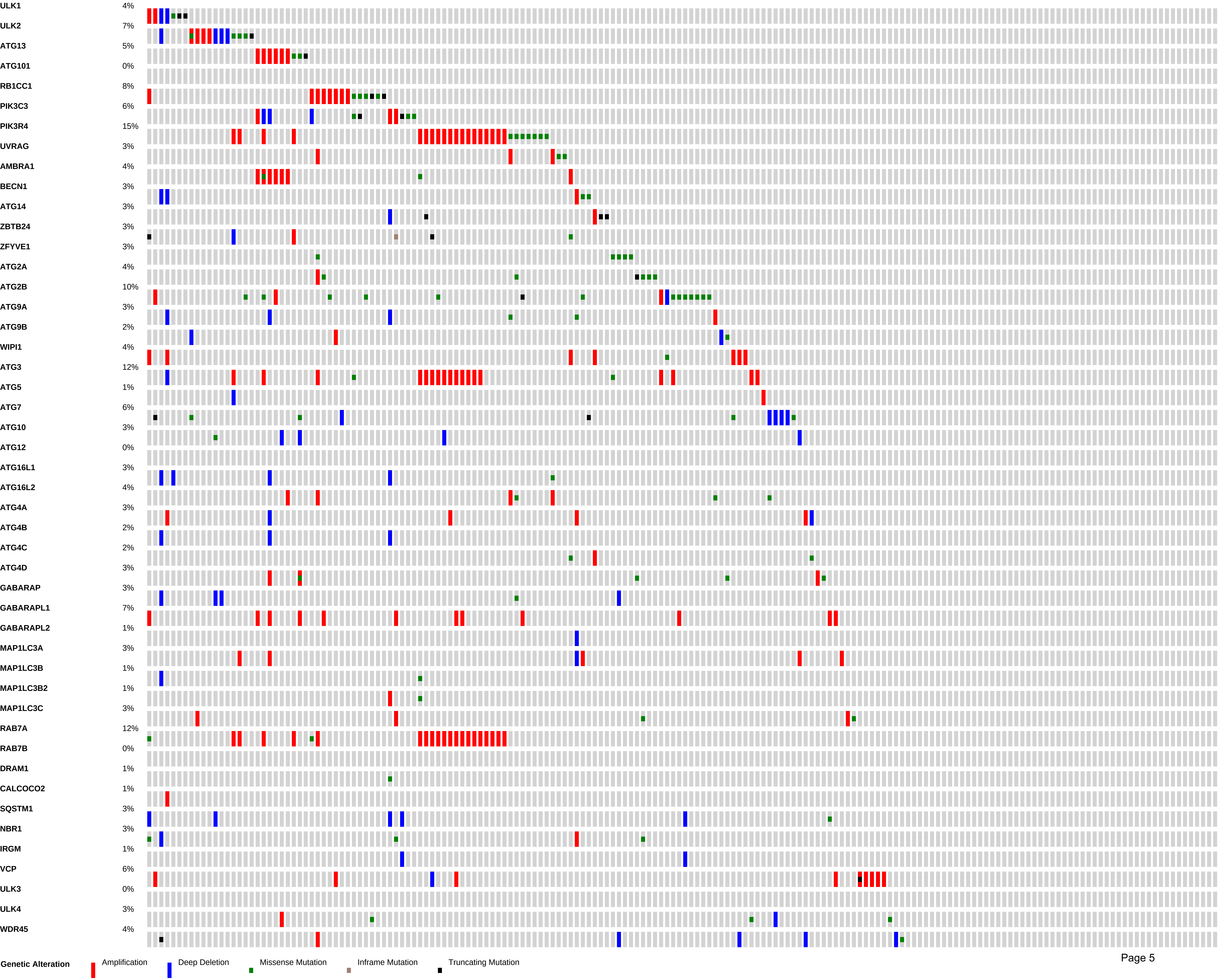
ULK1 4%
ULK2 7%
ATG13 5%
ATG101 0%
RB1CC1 8%
PIK3C3 6%
PIK3R4 15%
UVRAG 3%
AMBRA1 4%
BECN1 3%
ATG14 3%
ZBTB24 3%
ZFYVE1 3%
ATG2A 4%
ATG2B 10%
ATG9A 3%
ATG9B 2%
WIPI1 4%
ATG3 12%
ATG5 1%
ATG7 6%
ATG10 3%
ATG12 0%
ATG16L1 3%
ATG16L2 4%
ATG4A 3%
ATG4B 2%
ATG4C 2%
ATG4D 3%
GABARAP 3%
GABARAPL1 7%
GABARAPL2 1%
MAP1LC3A 3%
MAP1LC3B 1%
MAP1LC3B2 1%
MAP1LC3C 3%
RAB7A 12%
RAB7B 0%
DRAM1 1%
CALCOCO2 1%
SQSTM1 3%
NBR1 3%
IRGM 1%
VCP 6%
ULK3 0%
ULK4 3%
WDR45 4%
Genetic Alteration
Amplification
Deep Deletion
Missense Mutation
Inframe Mutation
Truncating Mutation

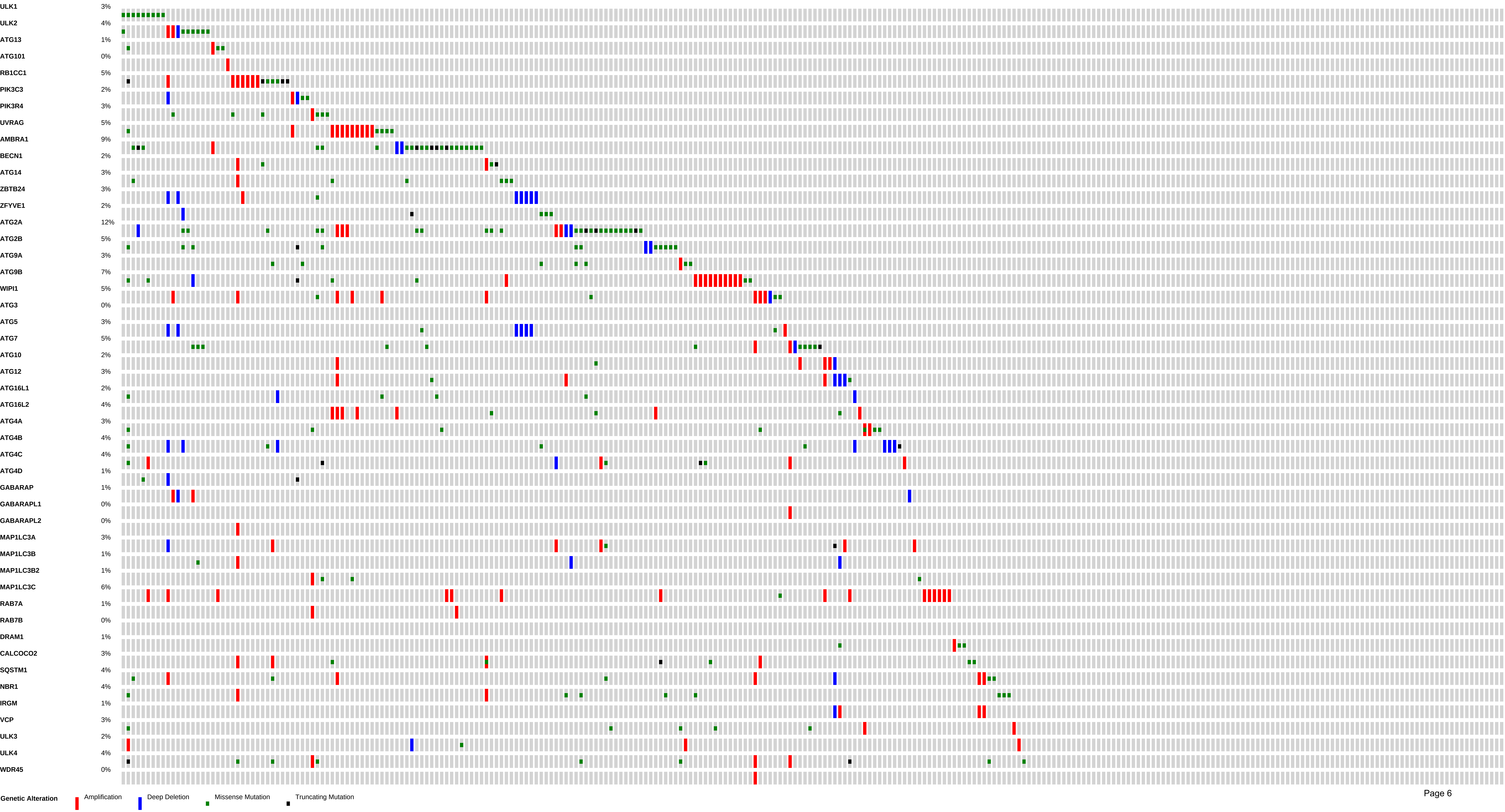

ULK1 3%
ULK2 4%
ATG13 1%
ATG101 0%
RB1CC1 5%
PIK3C3 2%
PIK3R4 3%
UVRAG 5%
AMBRA1 9%
BECN1 2%
ATG14 3%
ZBTB24 3%
ZFYVE1 2%
ATG2A 12%
ATG2B 5%
ATG9A 3%
ATG9B 7%
WIPI1 5%
ATG3 0%
ATG5 3%
ATG7 5%
ATG10 2%
ATG12 3%
ATG16L1 2%
ATG16L2 4%
ATG4A 3%
ATG4B 4%
ATG4C 4%
ATG4D 1%
GABARAP 1%
GABARAPL1 0%
GABARAPL2 0%
MAP1LC3A 3%
MAP1LC3B 1%
MAP1LC3B2 1%
MAP1LC3C 6%
RAB7A 1%
RAB7B 0%
DRAM1 1%
CALCOCO2 3%
SQSTM1 4%
NBR1 4%
IRGM 1%
VCP 3%
ULK3 2%
ULK4 4%
WDR45 0%
Genetic Alteration
Amplification
Deep Deletion
Missense Mutation
Truncating Mutation

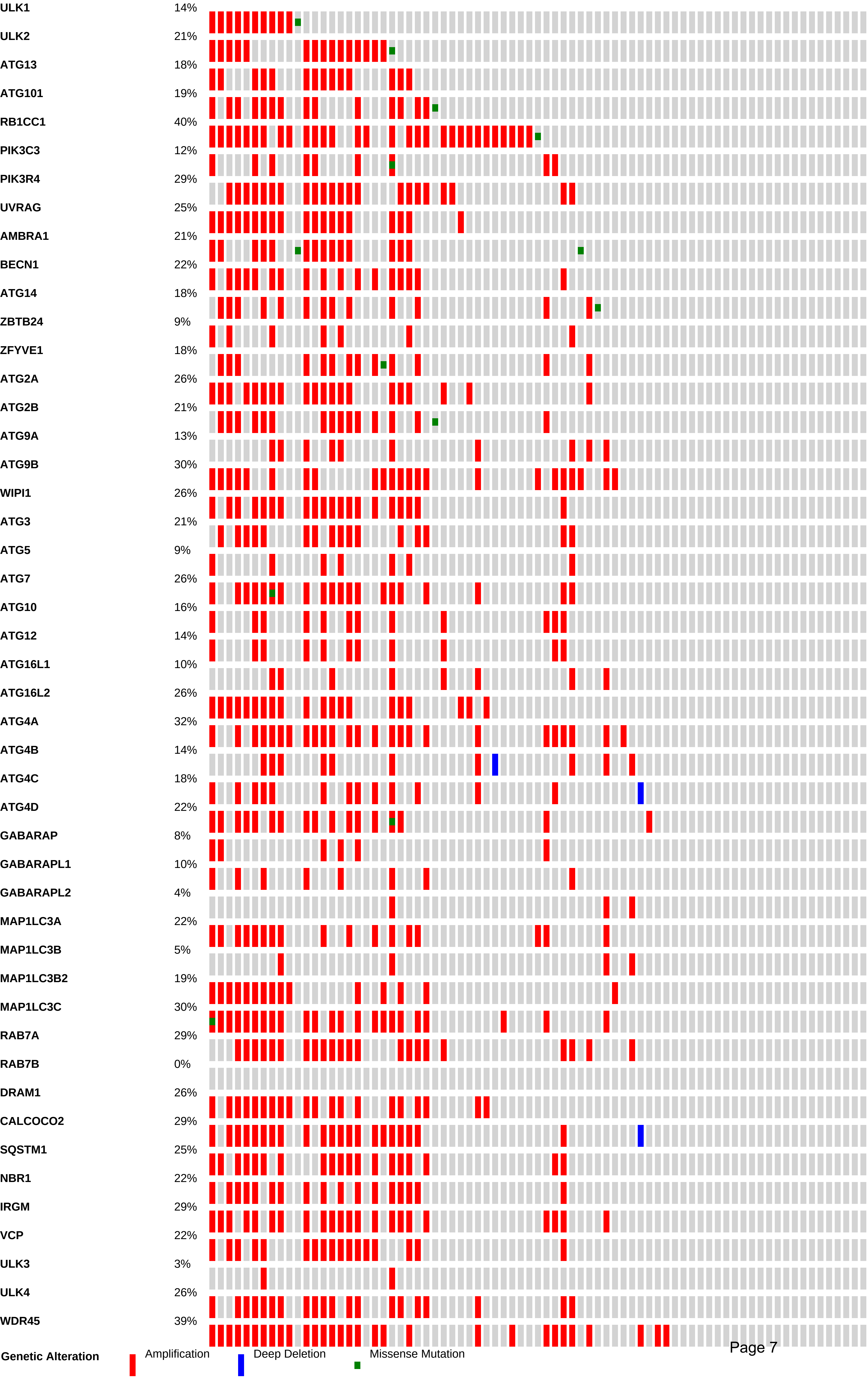
ULK1 14%
ULK2 21%
ATG13 18%
ATG101 19%
RB1CC1 40%
PIK3C3 12%
PIK3R4 29%
UVRAG 25%
AMBRA1 21%
BECN1 22%
ATG14 18%
ZBTB24 9%
ZFYVE1 18%
ATG2A 26%
ATG2B 21%
ATG9A 13%
ATG9B 30%
WIPI1 26%
ATG3 21%
ATG5 9%
ATG7 26%
ATG10 16%
ATG12 14%
ATG16L1 10%
ATG16L2 26%
ATG4A 32%
ATG4B 14%
ATG4C 18%
ATG4D 22%
GABARAP 8%
GABARAPL1 10%
GABARAPL2 4%
MAP1LC3A 22%
MAP1LC3B 5%
MAP1LC3B2 19%
MAP1LC3C 30%
RAB7A 29%
RAB7B 0%
DRAM1 26%
CALCOCO2 29%
SQSTM1 25%
NBR1 22%
IRGM 29%
VCP 22%
ULK3 3%
ULK4 26%
WDR45 39%
Genetic Alteration
Amplification
Deep Deletion
Missense Mutation

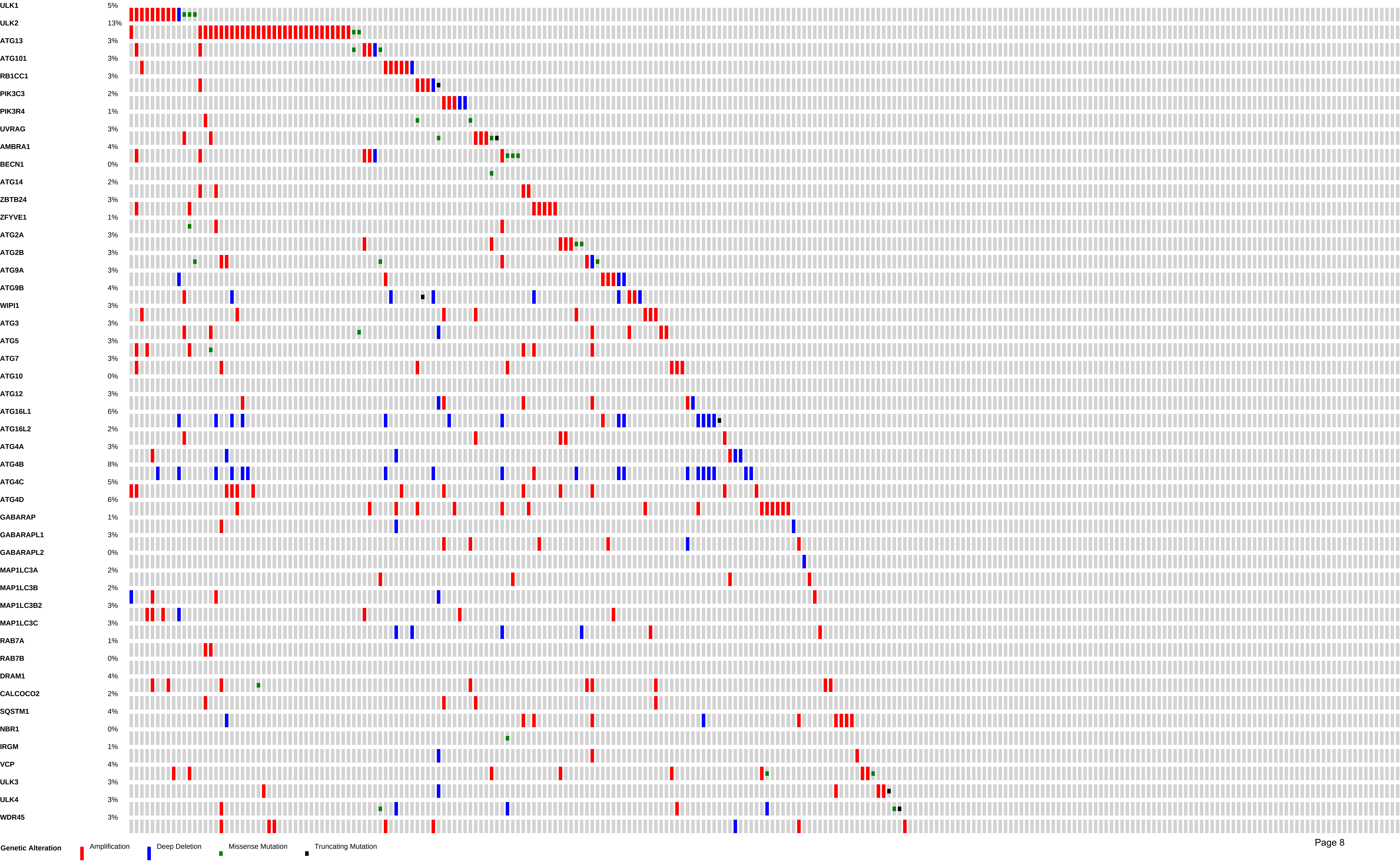
ULK1 5%
ULK2 13%
ATG13 3%
ATG101 3%
RB1CC1 3%
PIK3C3 2%
PIK3R4 1%
UVRAG 3%
AMBRA1 4%
BECN1 0%
ATG14 2%
ZBTB24 3%
ZFYVE1 1%
ATG2A 3%
ATG2B 3%
ATG9A 3%
ATG9B 4%
WIPI1 3%
ATG3 3%
ATG5 3%
ATG7 3%
ATG10 0%
ATG12 3%
ATG16L1 6%
ATG16L2 2%
ATG4A 3%
ATG4B 8%
ATG4C 5%
ATG4D 6%
GABARAP 1%
GABARAPL1 3%
GABARAPL2 0%
MAP1LC3A 2%
MAP1LC3B 2%
MAP1LC3B2 3%
MAP1LC3C 3%
RAB7A 1%
RAB7B 0%
DRAM1 4%
CALCOCO2 2%
SQSTM1 4%
NBR1 0%
IRGM 1%
VCP 4%
ULK3 3%
ULK4 3%
WDR45 3%
Genetic Alteration
Amplification
Deep Deletion
Missense Mutation
Truncating Mutation

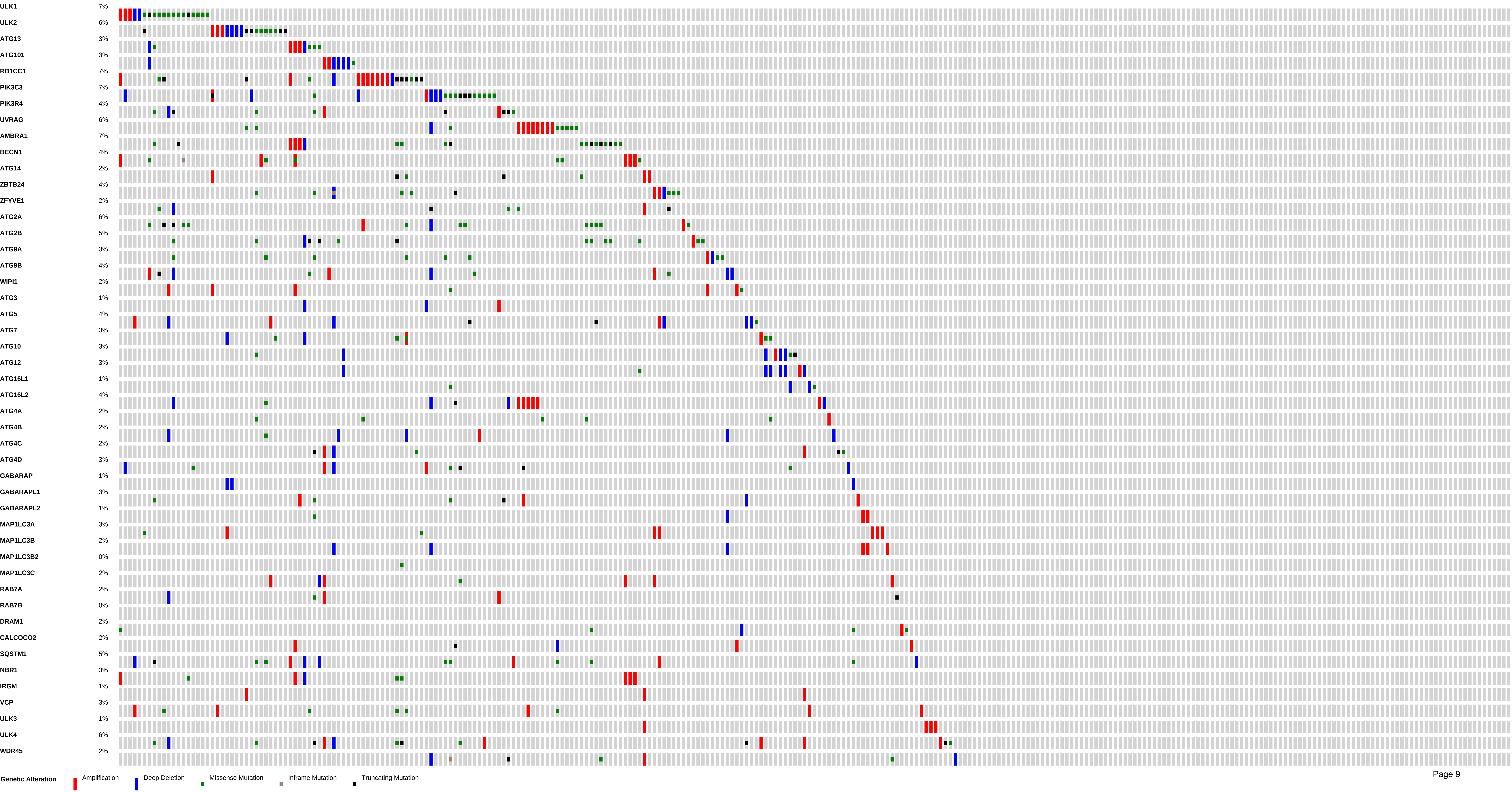
ULK1 7%
ULK2 6%
ATG13 3%
ATG101 3%
RB1CC1 7%
PIK3C3 7%
PIK3R4 4%
UVRAG 6%
AMBRA1 7%
BECN1 4%
ATG14 2%
ZBTB24 4%
ZFYVE1 2%
ATG2A 6%
ATG2B 5%
ATG9A 3%
ATG9B 4%
WIPI1 2%
ATG3 1%
ATG5 4%
ATG7 3%
ATG10 3%
ATG12 3%
ATG16L1 1%
ATG16L2 4%
ATG4A 2%
ATG4B 2%
ATG4C 2%
ATG4D 3%
GABARAP 1%
GABARAPL1 3%
GABARAPL2 1%
MAP1LC3A 3%
MAP1LC3B 2%
MAP1LC3B2 0%
MAP1LC3C 2%
RAB7A 2%
RAB7B 0%
DRAM1 2%
CALCOCO2 2%
SQSTM1 5%
NBR1 3%
IRGM 1%
VCP 3%
ULK3 1%
ULK4 6%
WDR45 2%
Genetic Alteration
Amplification
Deep Deletion
Missense Mutation
Inframe Mutation
Truncating Mutation

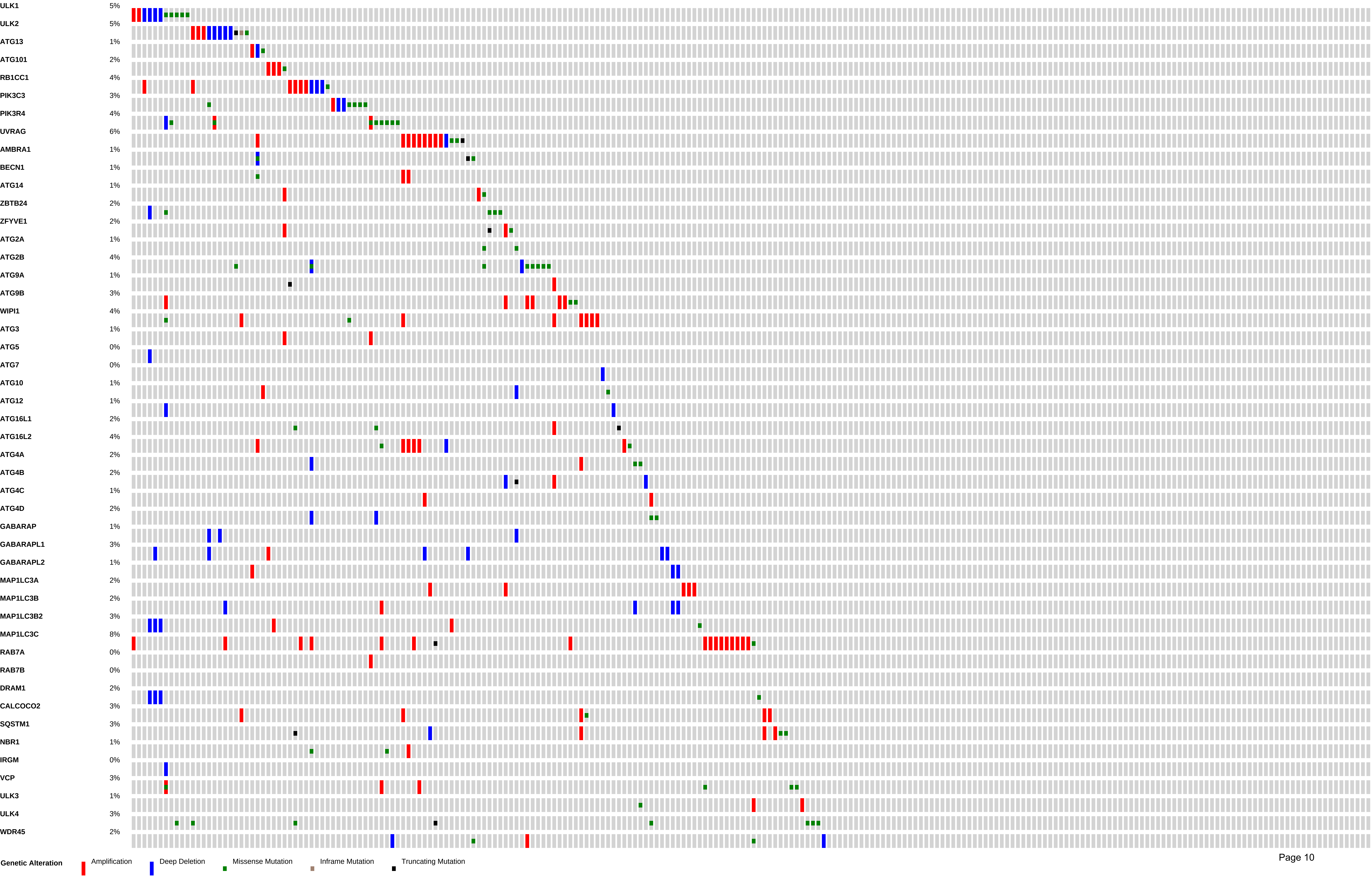
ULK1 5%
ULK2 5%
ATG13 1%
ATG101 2%
RB1CC1 4%
PIK3C3 3%
PIK3R4 4%
UVRAG 6%
AMBRA1 1%
BECN1 1%
ATG14 1%
ZBTB24 2%
ZFYVE1 2%
ATG2A 1%
ATG2B 4%
ATG9A 1%
ATG9B 3%
WIPI1 4%
ATG3 1%
ATG5 0%
ATG7 0%
ATG10 1%
ATG12 1%
ATG16L1 2%
ATG16L2 4%
ATG4A 2%
ATG4B 2%
ATG4C 1%
ATG4D 2%
GABARAP 1%
GABARAPL1 3%
GABARAPL2 1%
MAP1LC3A 2%
MAP1LC3B 2%
MAP1LC3B2 3%
MAP1LC3C 8%
RAB7A 0%
RAB7B 0%
DRAM1 2%
CALCOCO2 3%
SQSTM1 3%
NBR1 1%
IRGM 0%
VCP 3%
ULK3 1%
ULK4 3%
WDR45 2%
Genetic Alteration
Amplification
Deep Deletion
Missense Mutation
Inframe Mutation
Truncating Mutation

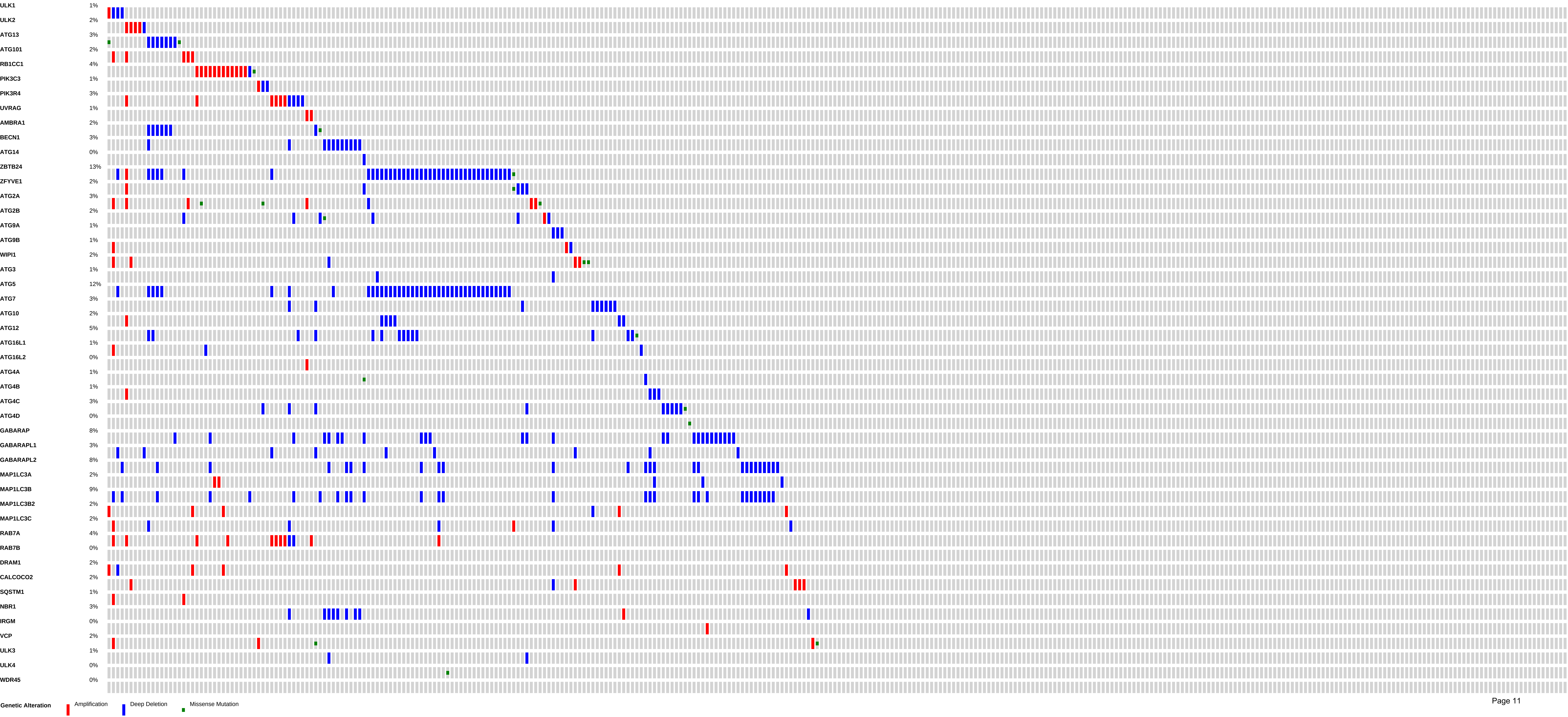

ULK1 1%
ULK2 2%
ATG13 3%
ATG101 2%
RB1CC1 4%
PIK3C3 1%
PIK3R4 3%
UVRAG 1%
AMBRA1 2%
BECN1 3%
ATG14 0%
ZBTB24 13%
ZFYVE1 2%
ATG2A 3%
ATG2B 2%
ATG9A 1%
ATG9B 1%
WIPI1 2%
ATG3 1%
ATG5 12%
ATG7 3%
ATG10 2%
ATG12 5%
ATG16L1 1%
ATG16L2 0%
ATG4A 1%
ATG4B 1%
ATG4C 3%
ATG4D 0%
GABARAP 8%
GABARAPL1 3%
GABARAPL2 8%
MAP1LC3A 2%
MAP1LC3B 9%
MAP1LC3B2 2%
MAP1LC3C 2%
RAB7A 4%
RAB7B 0%
DRAM1 2%
CALCOCO2 2%
SQSTM1 1%
NBR1 3%
IRGM 0%
VCP 2%
ULK3 1%
ULK4 0%
WDR45 0%
Genetic Alteration
Amplification
Deep Deletion
Missense Mutation

A

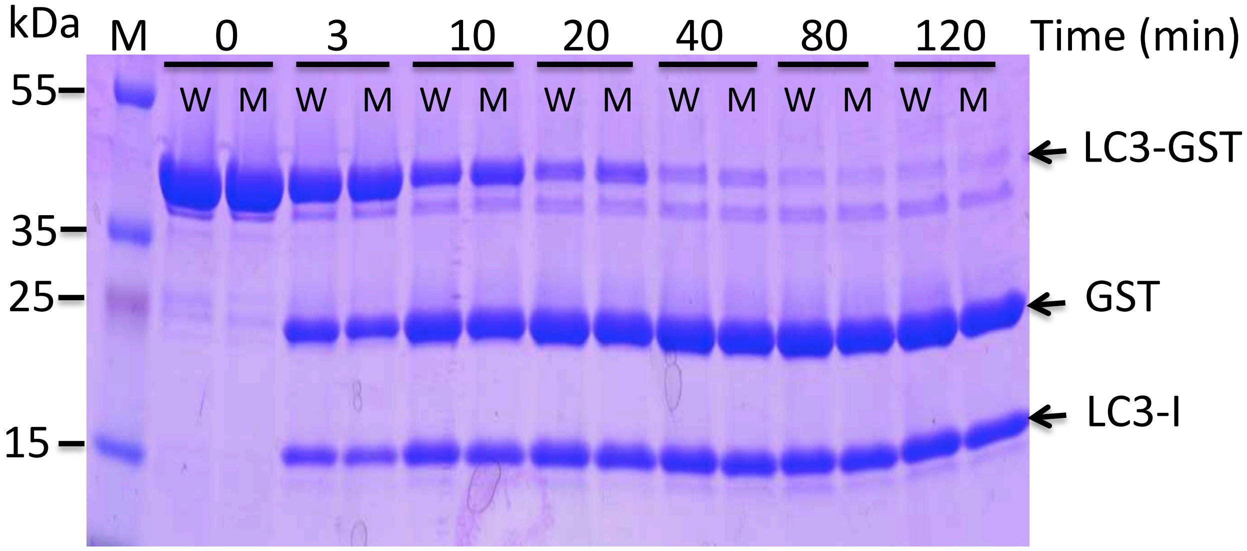

B

Percentage of the remaining substrate (LC3A-GST or LC3AR70H-GST) after digestion with ATG4B

Digestion time (min)	0	3	10	20	40	80	120
**LC3AWT**	100	48.38 ± 3.24	24.97 ± 1.56	13.56 ± 1.11	6.54 ± 0.38	2.13 ± 1.14	1.56 ± 0.19
**LC3AR70H**	100	51.97 ± 1.13	29.39 ± 3.44	16.73 ± 0.59	7.58 ± 0.29	2.74 ± 0.78	1.80 ± 0.71

Supplementary Figure: *In vitro* proteolysis assay (A) SDS-PAGE analysis of the digestion of MAP1LC3A-GST and and MAP1LC3A70H-GST at 0.525 mg/ml by ATG4B at 0.00133 mg/ml at 37°C for 3 to 120 min. Arrows indicate where the substrate LC3-GST and the products GST and LC3-I on SDS-PAGE. W and M represent wild type and mutant. (B) The percentage of the remaining substrate (LC3A-GST or LC3AR70H-GST) after digestion with ATG4B was calculated from 3 independent experiments and the values are shown as ± SE.

Primer Sequences Used for the Plasmid Cloning		
Name of Primer	Primer Sequence	Tm (°C)
LC3A_NcoI	5’- AAA CCA TGG CAC CCT CAG ACC GGC CTT TCA AGC AG-3’	62
LC3A_R	5’- GAA GCC GAA GGT TTC CTG GGA GGC-3’	
SmaI-GST	5’-CCC GGG ATG TCC CCT ATA CTA GGT TAT TGG-3’	61
GST_HIndIII_R	5'- TTT AAG CTT AGG TTT TCA CCG TCA TCA CCG AAA-3'	
LC3AR70H_F	5’- ACC TGC AGC TGA ACC CCA CGC AG-3’	62.5
LC3AR70H_R	5’-GGC GCC GGA TGA TCT TGA CCA ACT C -3’	

Supplementary Figure: Primer sequences used in this study.
